# Cost and economic evidence for asset-based approaches to health improvement and their evaluation methods: a systematic review

**DOI:** 10.1186/s12889-024-18231-4

**Published:** 2024-03-15

**Authors:** Alice Wreford, Linda Birt, Jennifer A. Whitty, Sarah Hanson, Susan Conquer, Adam P. Wagner

**Affiliations:** 1 University of East Anglia, Norwich, UK; 2 NIHR Applied Research Collaboration (ARC) East of England (EoE) Health Economics and Prioritisation in Health and Social Care Theme, Cambridge, UK; 3 School of Healthcare, University of Leicester, Leicester, UK; 4 Evidera, The Ark, 2nd floor, 201 Talgarth Road, London, W6 8BJ UK; 5 Healthwatch Suffolk, Ipswich, UK

**Keywords:** Asset, Community development, Economic evaluation, Costing, Social connection, Equity, Marginalised, Inequalities

## Abstract

**Background:**

Asset-based approaches (ABAs) tackle health inequalities by empowering people in more disadvantaged communities, or targeted populations, to better utilise pre-existing local community-based resources. Using existing resources supports individuals to better manage their own health and its determinants, potentially at low cost. Targeting individuals disengaged with traditional service delivery methods offers further potential for meaningful cost-savings, since these people often require costly care. Thus, improving prevention, and management, of ill-health in these groups may have considerable cost implications.

**Aim:**

To systematically review the extent of current cost and economic evidence on ABAs, and methods used to develop it.

**Methods:**

Search strategy terms encompassed: i) costing; ii) intervention detail; and iii) locality. Databases searched: Medline, CENTRAL and Wed of Science. Researchers screened 9,116 articles. Risk of bias was assessed using the Critical Appraisal Skills Programme (CASP) tool. Narrative synthesis summarised findings.

**Results:**

Twelve papers met inclusion criteria, representing eleven different ABAs. Within studies, methods varied widely, not only in design and comparators, but also in terms of included costs and outcome measures. Studies suggested economic efficiency, but lack of suitable comparators made more definitive conclusions difficult.

**Conclusion:**

Economic evidence around ABAs is limited. ABAs may be a promising way to engage underserved or minority groups, that may have lower net costs compared to alternative health and wellbeing improvement approaches. ABAs, an example of embedded services, suffer in the context of economic evaluation, which typically consider services as mutually exclusive alternatives. Economics of the surrounding services, mechanisms of information sharing, and collaboration underpin the success of assets and ABAs. The economic evidence, and evaluations in general, would benefit from increased context and detail to help ensure more nuanced and sophisticated understanding of the economics of ABAs. Further evidence is needed to reach conclusions about cost-effectiveness of ABAs.

**Supplementary Information:**

The online version contains supplementary material available at 10.1186/s12889-024-18231-4.

## Background

### Asset based approaches to health

In the United Kingdom (UK) a growing and ageing population, and increasing burden on health services, is leading to an emphasis on prevention, integration of services and supporting people to manage their own health [1]. Historically, approaches to improve population health have utilised a *‘deficit model’*: focusing on identifying the problems and needs of the population that require costly curative services [2–4]. This undermines the role that individuals and communities can play as active participants to create, acquire and maintain their own health [2]. The NHS Long Term Plan (2019) calls for a shift towards a pro-active model for health and well-being, whereby society values and tries to enable a state of complete physical, mental and social well-being, as opposed to waiting for individuals to reach ill-health before receiving support and treatment [5, 6]. This recognising that positive health and social outcomes can be achieved more successfully and efficiently if there is a shift away from a ‘doing to’ culture (whereby individuals are treated once they become unwell) to one that respects meaningful and ‘preventative’ social change (where individuals are supported and empowered to make healthier choices, promoting continued good health) [7].

‘Assets’ and ‘asset-based approaches’ (ABAs) aim to identify and utilise health promoting or protective factors that are most likely to lead to higher degrees of overall health and wellbeing, achievement, and sustainability [2]. ‘Assets’ are community resources, and can refer to financial resources, physical infrastructure, facets of social capital or individual capabilities [8]. Assets operate across multiple levels, for example enabling self-esteem and resilience at the individual-level, supportive friendship and peer networks at community-level, and provision of positive environmental and organisational resources to promote health and well-being at structural-levels [9]. ABAs build on the skills of local people, the power of local associations and the supportive functions of institutions and services, to build stronger, more sustainable communities [10]. ABAs are considered a subset of community-based interventions, distinctly implementing interventions which aim to build capacity, engagement, knowledge and/or resources within a defined community asset e.g., local churches, existing community groups, local parks or hyperlocal geographical areas. They make visible and value the skills, knowledge, connections and potential in a community [11].

Generally speaking, health and wellbeing services suffer from disparity in uptake and provision, such that uptake is socially patterned, and services are more likely to be successfully accessed by affluent groups—termed the ‘*inverse prevention law’* [12, 13]. Therefore, preventative health interventions may not reach the most disadvantaged and those furthest from reaching their full health potential [12]. Consequentially, preventative services may benefit from ABAs. One of the main advantage of ABAs, beyond standard community interventions, is that they can engage: i) members of society who are more likely to be disengaged with traditional methods of service delivery; and ii) specific populations (e.g. cancer survivors). They do this by tapping into existing community networks, making use of existing rapport and trusted connections, to facilitate health-related conversations and information sharing which otherwise may not occur. Therefore, equitable community-based development may be best supported through ABAs [14, 15]. Further, building on existing assets potentially reduces high-level start-up costs, such as reducing the need for highly trained staff to address participant care needs, alongside delivering engagement and implementing strategies. In ABAs, individuals working in established groups are typically already well-versed at meeting their participants’ needs [16]. Since there are reduced start-up costs, and engagement is expected to be high, from an economic perspective, ABAs offer a potentially efficient approach to delivering benefits for participants.

### Economic evaluation of ABAs

Decision-makers must routinely make choices about how to prioritise public health problems and related interventions within limited budgets and resources. In making such choices, decision-makers can benefit by knowing the financial resources required to implement each intervention and how money invested compares to outcomes achieved [17]. Economic comparisons are challenging in the context of: i) comparing one ABA to another ABA; and ii) comparing ABAs to other types of health and social care interventions. To date, it is unclear what economic evidence has been collected in the context of ABAs and what methods have been used to generate this evidence.

Well-established frameworks, known as economic evaluations (EEs), inform decision-makers about the comparative costs and outcomes (or ‘benefits’) of a range of mutually exclusive courses of action for health and social care. From this definition, it is worth emphasising that EEs do not exclusively focus on costs—outcomes/benefits are crucially important too. Typically, EEs involve exploring which option will maximise health and social care outcomes, most commonly in the form of quality-adjusted life years (QALYs) that seek to capture impacts on life extension and its quality, subject to the constraints of the health sector budget. Public health and social care interventions, including ABAs, may be considered complex interventions, in that they offer flexibility in intervention delivery and individual-level variability in outcomes (e.g. improving choice, access and participation in healthcare, education, housing, employment, social activity and personal care) [18]. Complex intervention research “goes beyond asking whether an intervention works in the sense of achieving its intended outcome – to asking a broader range of questions (e.g., identifying what other impact it has… taking account of how it interacts with the context in which it is implemented, how it contributes to system change…)” (P.1, [19]). Therefore, while EEs may be appropriate to assess the value of ABAs, the use of health-focused outcomes, such as QALYs, are unlikely to capture their full impacts. Wider wellbeing outcomes, often measured as wellbeing adjusted life years (WALYs or WELBYs), have received growing interest in the evaluation of public health interventions [20, 21]. While these measures purport to measure and value a broader range of dimensions of health and wellbeing, they are generally viewed as sub-optimal for evaluating complex interventions where the full spectrum of benefits extends well beyond health [22].

Beyond outcome measurement, the EE techniques that typically utilise QALYs (e.g. cost-utility analyses) generally focus on the health care sector (health perspective), often again ‘missing’ some of the important wider societal impacts. A wider societal perspective may be adopted, capturing costs across sectors (e.g. beyond health) with the aim of maximising welfare gain to society [23]. However, this approach is still limited, with impact primarily affecting the ‘cost side’ of the equation. EEs which seek to capture the full range of health and non-health costs and benefits across different sectors (e.g. cost consequence analysis, cost benefit analysis or multicriteria decision analysis) may be more relevant in this context. However, these approaches require multidimensional datasets, potentially complex modelling approaches, and offer an opportunity for inappropriate interpretation of results if the evaluation is not appropriately designed for the decision context [24].

Importantly, *health equity,* a key objective in public health policy, is rarely captured in economic evaluations [25]. With the increasing awareness of inequity in health improvement, it is essential that health economic methodologies develop to capture this under-reported outcome. Health economics is regularly centred in the quasi-egalitarian value judgement that *‘a QALY is a QALY’*; where “QALYs are equally weighted and the health outcome is worth the same no matter how it is achieved, or by whom it accrues” (P.231, [25]). This assumption conflicts with an increasing focus on improving health equity and reaching under-served populations. As ABAs seek to reach those furthest from their health potential, there is opportunity for intervention impact to be greater than it would be for the general population. Specifically in the context of prevention, health improving behavioural shifts (e.g. increased use of health services) may be more impactful when initial behaviours were more harmful (i.e. those who rarely access services may see greater health and wellbeing improvements than those who already regularly engage with services). More broadly, the formerly prevalent view of *health gain maximisation* – where the objective is primarily to maximise total health benefit [26, 27] – may be undervaluing some interventions as it does not capture the entire impact and value of engaging disengaged individuals to effectively manage their own health. Thus, one might question the applicability of non-targeted health maximising of QALYs in the community care setting – perhaps it does matter by whom it is accrued, providing an argument for weighting cost per QALY by who is benefitting. Additionally, others have noted that allocation of health care resources solely based on health maximation can lead to discrimination against certain groups [28].

A challenge in evaluating ABAs is establishing a suitable alternative to compare the ABA to. In the case of novel asset-based interventions, which are unique in their localised social structures, appropriate comparators are not always clear. The intervention may be compared to the ‘absence of the intervention’ and/ or ‘standard care’. However collecting data for these alternatives is challenging with ABA interventions, since they often involve whole communities in a specific locale (e.g. potential lack of unaffected ‘control’ individuals) and often utilise information sharing and capacity building (consequently statistical ‘contamination’ is likely between intervention and control groups—discussed further later). Absence of a suitable alternative precludes a full EE, and more generally can make it difficult to robustly determine the impact of an intervention.

To better support development and evaluation in complex intervention settings, the National Institute of Health Research and the UK Medical Research Council Guidance commissioned a framework [originally developed 2006; last updated 2021] [19]. This guidance is well positioned for ABA development as it recognises the context specific complexity, framing interventions as events in a wider system. The framework appreciates the pragmatic embedding of services, and its conflict with the assumptions of mutual exclusivity of interventions required for current EE methods – suggesting supporting qualitative studies may be useful to support interpretation of EEs [19]. The framework highlights crucial aspects of evaluation design, including appropriate: comparator; choice of outcome measure or evidence of change; study design; and, costs (including implementation and set-up costs) [19].

Recent developments in the guidance have focused on giving attention to properties of the intervention itself, such as: range of behaviours targeted; expertise and skills required by those delivering and receiving the intervention; number of groups; community setting; and, level of flexibility of the intervention or its components. Appropriate design of complex interventions allows for flexibility in implementation, whilst maintaining the integrity of the core intervention components. This recognises that the ‘same’ ABA applied in two different settings may look different as they appropriately and intentionally adapt to the community setting and target demographic needs. These flexible properties of the intervention have implications for many aspects of its evaluation, and subsequent interpretation. Thus, intervention detail will be considered within this review.

## Aims

To make best use of scarce public budgets, it is important to understand the current cost and economic evidence base for ABAs. Further, to inform future research, we want to investigate the methods used to create the current evidence. Thus, this systematic review seeks to address:

i) What designs, methods, and outcomes measures have been used to produce cost and economic evidence?
ii) What is the accrued evidence for the cost and economic impact of ABAs?

## Methods

The protocol for this systematic review has been registered in PROSPERO (CRD42021236548). The Preferred Reporting Items for Systematic Reviews and Meta-Analyses (PRISMA) guidelines were followed [29].

### Patient and public involvement (PPI) and stakeholder contribution

Given the broad array of potential community assets, and the flexibility of ABAs, it is challenging to outline a formal or universal definition. Hence, PPI partners and stakeholders were closely involved in defining and categorising ABAs, development, and finalisation of the search terms and strategy, and evaluating the quality of evidence found (e.g., reflecting on the range of outcome measures used and their suitability). They brought specific experience to the review through their roles as a local authority commissioning manager and local Healthwatch co-production facilitator [‘Healthwatch’ is a health and social care champion in England, which ensures service providers and decisions makers listen to public feedback, with the aim to improve care (https://www.healthwatch.co.uk/)]. Each was familiar with ABAs prior to involvement in this study. This level of partner involvement was essential in the exploratory work to help ensure the questions asked of the literature and focus of analysis resonated with public health practice.

### Search strategy

The search strategy consisted of three broad components capturing terms associated with: i) costing; ii) intervention detail; and iii) locality. The search strategy was developed with PPI contribution (outlined in Sect. "Inclusion and exclusion criteria"). The second component (intervention detail) received careful attention, helping to ensure eligible papers where the intervention was not explicitly termed or identified as an ABA were captured. Terms included: ‘asset’; 'coproduction’; ‘community development’; ‘capabilities’; ‘resilience’; ‘salutogenesis’; ‘self-care’; ‘participatory approach’; ‘peer support’; ‘intervention’; ‘social prescription’; ‘health promotion’; ‘lay worker’; ‘wellbeing officer’; ‘community health worker’; ‘capacity’; and ‘upskill’. Complete search terms are given in Supplementary Material 1.

Electronic bibliographic databases [MEDLINE (via PubMED); Cochrane Centre Register of Controlled Trials (CENTRAL); and Web of Science] were searched on 07.03.23, with no publication date restrictions.

### Inclusion and exclusion criteria

The following inclusion and exclusion criteria were employed.

Inclusion – all required:

- Interventions categorised as ABA (following our above definition), even when not explicitly named or labelled as such.
- Studies which evaluated the *‘cost’,* or ‘*costs and benefits’* relating to at least one ABA.
- Interventions delivered by non-clinical professionals – e.g. community members, community service staff. Interventions may involve a training component, supportive role or be co-produced with health practitioners.
- Peer reviewed papers only.
- English language only.

Exclusion, where any of the following apply:

- Interventions delivered in clinical settings (i.e. interventions centred in hospital or General Practices).
- Community setting not detailed.
- Interventions administered via telephone or cyber communication, unbound by geographical constraint.
- Interventions delivered exclusively by a clinical professional.
- Studies which do not report cost(s) associated with the intervention.

### Study selection

Database search results were extracted to a citation manager (Endnote X9) where duplicates were identified and removed. Study details (including title and abstracts) were uploaded to web-based software Rayyan (https://www.rayyan.ai/) for screening. Article abstracts were shared between two researchers (AW & LB). Within Rayyan, each reviewer recorded for each article whether it was rejected or put forward for stage two review.

Articles selected for full-text review were independently compared to the inclusion/ exclusion criteria by two reviewers (AW & LB) to finally determine their inclusion. Where these reviewers disagreed, a third reviewer (JAW) arbitrated.

### Data extraction

A data extraction table was developed in MS Excel (Supplementary Material 2), capturing 20 study characteristics, and populated by AW.

Included studies were categorised into three groups:

i) Reporting only implementation and running costs (IRC);
ii) Reporting implementation and running costs AND health and/or social care related costs (IRHSC);
iii) Including an economic evaluation (EE) (thus a joint comparison of costs and benefits comparing the ABA to suitable comparator).

Studies categorised to i) IRC and ii) IRHSC did not require a study comparator.

### Quality of reporting assessment

The Critical Appraisal Skills Programme (CASP) quality appraisal tool (reported in full in Supplementary Material 3) was used to assess the risk of bias. CASP was selected as it can be used to assess a wide range of both qualitative and quantitative studies, including, randomised controlled trials, cohort studies and economic evaluations [30]. For each study, a CASP checklist relevant to its study type was selected.

## Results

### Literature search and evaluation for study inclusion

**Fig. 1:**
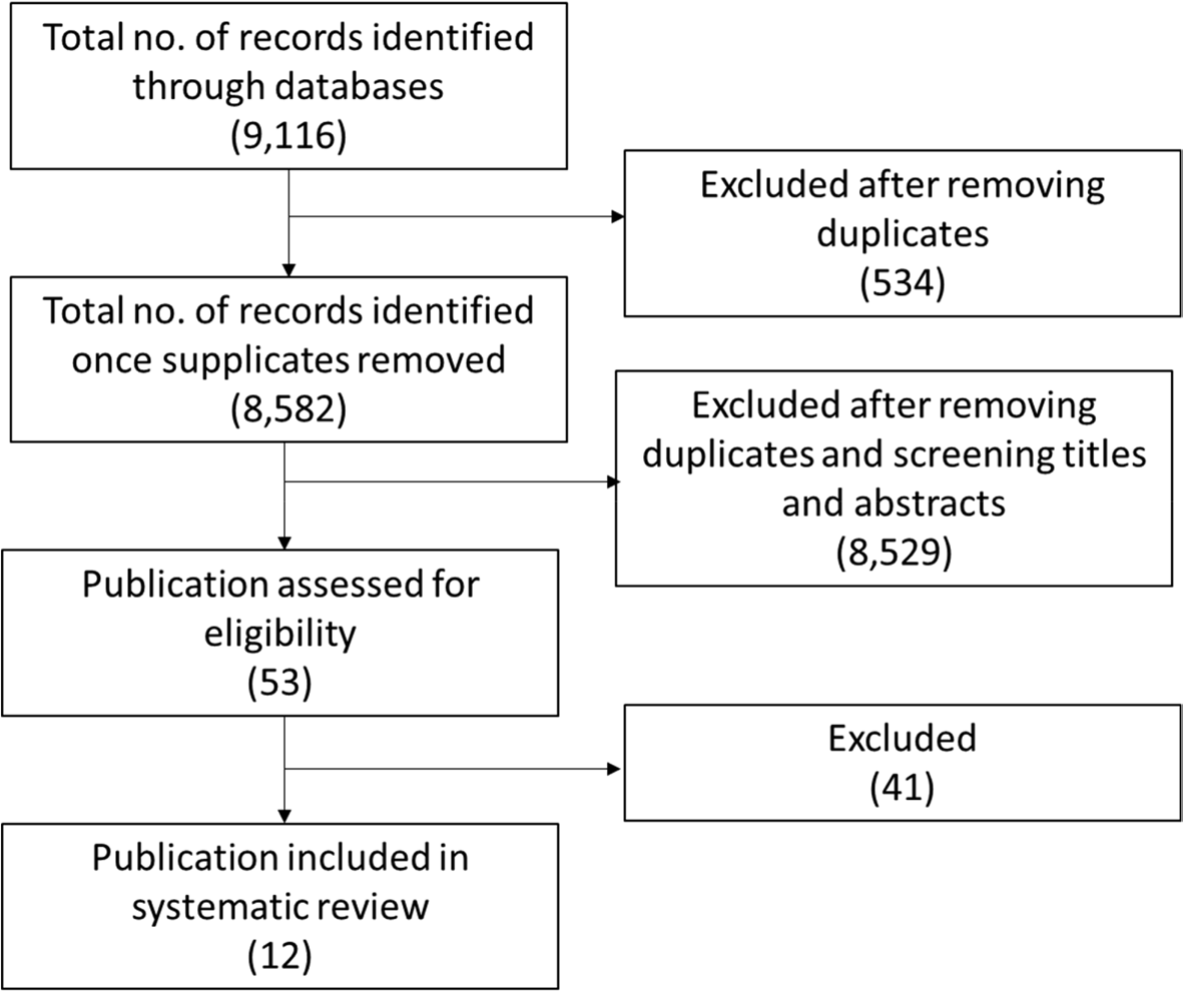
PRISMA diagram for search process

A summary of the process of review and selection is given in the PRISMA flow diagram (Fig. 1).

Searches yielded a total of 9,116 results, of which 534 were identified as duplicates. Subsequently, 8,582 abstracts were screened. Of the screened abstracts, 8,529 studies were excluded, with unanimous (100%) agreement between primary reviewers (AW & LB). The 53 remaining papers underwent full text review: 48 had their inclusion/exclusion status agreed between by the first two reviewers (AW & LB), with disagreement on the remaining five studies resolved by the third reviewer (JAW). Twelve publications were agreed for inclusion.

Studies were appraised using the CASP tools relevant to study type (Tables 1, 2 and 3).

**Table 1:** Results of CASP cohort study assessment

*Study*	*1*	*2*	*3*	*4*	*5a*	*5b*	*6a*	*6b*	*7*	*8*	*9*	*10*	*11*	*12*
*Kahn,USA, 2001* [31]	*Y*	*Y*	*Y*	*Y*	*Y*	*N*	*Y*	*Y*	*Cost per HIV infection averted far less than lifetime medical costs of managing HIV* *Cost depends on session numbers:* *1 session: $4,150 per HIV infection averted;* *12 sessions: $50,000 per HIV infection averted*	*Sensitivity analysis conducted*	*Y*	*Y*	*Y*	*Y*
*Mayer, USA, 2010* [32]	*Y*	*Y*	*Y*	*Y*	*Y*	*Y*	*Y*	*?*	*1 year follow-up, considered reasonable for context* *However, as preventative service, unclear if life-course duration should be considered*	*Reported non-significant (p* = *0.58) health care cost saving and difference in hospitalisation* *Participants had significantly lower comorbidity (major driver in hospitalisation) than non-participants*	*Y*	*Y*	*Y*	*Y*
*Eckermann, Australia, 2014* [33]	*Y*	*Y*	*Y*	*Y*	*Y*	*N*	*Y*	*Y*	*Successful health promotion program with* *high community network impact and* *return on investment in practice*	*No confidence intervals reported*	*Y*	*Y*	*Y*	*Y*

Key: *Y* Yes, *N* No, ? Can’t tell

**Table 2:** Results of CASP Randomised controlled trial assessment

Study	1	2	3	4a	4b	4c	5	6	7	8	9	10	11
Ellis-Hill, UK, 2019 [34]	Y	Y	Y	N	N	?	Y	Y	Y	Y	Y	Y	?
Krukowski, USA, 2013 [35]	Y	Y	?	N	N	?	Y	?	N	Y	Y	Y	Y
Gitlin, USA, 2012 [36]	Y	Y	Y	?	?	?	Y	Y	Y	Y	Y	Y	?
Pizzi, USA, 2014 [37]	Y	Y	Y	?	?	?	?	Y	Y	N	Y	Y	Y
Chung, USA, 2018 [38]	Y	Y	Y	N	N	?	Y	Y	Y	Y	Y	Y	N
Yeary, USA, 2020 [39]	Y	Y	?	N	N	?	Y	Y	Y	Y	Y	Y	?

Key: *Y* Yes, *N* No, ? Can’t tell

**Table 3:** Result of CASP economic evaluation checklist

Study	1	2	3	4	5	6	7	8	9	10	11
Stevens, UK, 2002 [40]	Y	Y	Y	Y	Y	?	Y	Y	Y	Y	Y
Wingate, UK, 2017 [41]	Y	Y	Y	Y	Y	Y	Y	Y	Y	Y	Y
Visram, UK, 2020 [42]	Y	Y	Y	Y	Y	Y	Y	Y	Y	Y	Y

Key: *Y* Yes, *N* No, ? Can’t tell

### Overview of studies

Twelve papers, representing eleven different ABAs, met the inclusion criteria: Pizzi 2014 [37] assesses the cost-effectiveness of the ABA reported in Gitlin 2012 [36]. Characteristics of included studies relate to the 11 ABAs, treating Pizzi 2014 and Gitlin 2012 as one study.

Most papers [*n* = 10] were published between 2010–2020, with two published in 2001–2002 [31, 40]. Papers were categorised to: reporting only implementation and running costs (IRC) [*n* = 1: [36]]; reporting implementation and running costs AND health and/or social care related costs (IRHSC) [*n* = 2: [32, 34]]; and including an economic evaluation (EE) [*n* = 9: [31, 33, 35, 37–42]]. Here forward, where counted, paper categories are indicated with subscripts.

The reported ABAs were delivered in the: USA [*n* = 7: n_IRC_ = 1 [36], n_IRHSC_ = 1 [32], n_EE_ = 5 [31, 35, 37–39]]; Australia [n_EE_ = 1 [33]]; and the UK [*n* = 4: n_IRHSC_ = 1 [34], n_EE_ = 3 [40–42]]. Among selected studies, only one labelled its intervention as an ABA [42], with others using phrases such as “delivered through community-based organisations” (P.2, [36]) and “engage with social networks and build social capital to enable community ownership” (P.104, [33]). Table 4 details phrases used to describe the ABAs among included papers.

**Table 4:** Included studies summary: populations, interventions, comparators

**Study**	**Population**	**Intervention aim**	**Intervention type**	**Intervention title**	**Intervention detail**	**Individuals delivering service**	**Community setting**	**Intervention labelled as ABA?** *If no, phrase used?*
**Reporting only implementation and running costs (IRC)**								
Gitlin, USA, 2012 [36]	African Americans; ≥ 55; English speaking; ‘cognitively intact’ (MMSE > 24), ≥ 5 on PHQ-9	Reduce depressive symptoms	Screening & education programme	Beat the Blues	Implement routine screening for depression and offer culturally sensitive coping (non-pharmacology) strategies	Senior centre care management staff	Senior centres	No; “Delivered through community-based organisations”
**Reporting implementation and running costs AND health and/or social care related costs (IRHSC)**								
Mayer, USA, 2010 [32]	Ling County residents; ≥ 65	Prevent disabilities, improve health and functioning in older adults	Education programme	Enhance Wellness	Wellness programme with health assessment, a tailored health plan and motivational support to achieve self-chosen goal	Hosting site or senior services staff	Community centres (predominately senior centres)	No; “connect clients with information and resources that help them address their personal health concerns”
Ellis-Hill, UK, 2019 [34]	Adults; up to 2 years post stoke	Improve mood of individuals post stroke	Peer support service	HeART of Stroke	Art and health group (10 × 2-h sessions over 14 weeks) + standard care	Artist-facilitated	Community centres	No; “creative approaches in health provision” “community arts and health group intervention”
**Including an economic evaluation (EE)**								
Kahn, USA, 2001 [31]	‘Gay and bisexual men’; 18–27	Reduce HIV infection	Education programme	Empowerment Project	Peer outreach with young gay men to encourage peers to engage in safer sex and recruit additional young gay men. Peer led 3x1 hour group meetings (involving discussions, exercise, and role-plays on issues related to HIV, and train and motivate outreach)	Young gay men in the community, with support of two behavioural intervention researchers	Universities, bars, community events	No; “community-level intervention”
Stevens, UK, 2002 [40]	Turkish community in Camden and Islington	Promote non-smoking as norm; reduce prevalence of smoking	Education programme	-	Theatre performance (written and performed by local Turkish community) and poster campaign	Turkish drama group: ‘Tiyatro Ala-Turka’	Local Turkish venues (café, advice and community)	No; “targeting specific groups for direct action”
Krukowski, USA, 2013 [35]	Adults ≥ 60; obese (BMI ≥ 30); no significant memory problems (Mini mental State Exam score ≥ 23)	Implement diabetes prevention program in high-risk group	Education programme	Lifestyle Education	‘Diabetes Prevention Programme’ adapted for group setting to support weight loss among high-risk group	Trained lay worker	Senior centres	No: “Programmes delivered by Lay Health Educators might support greater dissemination [in the community]”
Pizzi, USA, 2014 [37]	African Americans; ≥ 55; English speaking; cognitively intact (MMSE > 24), and ≥ 5 on PHQ-9	Reduce depressive symptoms	Screening and Education programme	Beat the Blues	Implement routine screening for depression and offer culturally sensitive coping (non-pharmacology) strategies	Senior centre care management staff	Senior centres	No; “Delivered through community-based organisations”
Eckermann, Australia, 2014 [33]	School students; 8–12; school years 3–6	Improve lifestyle behaviours, food choices and eating habits	Education programme	Stephanie Alexander Kitchen Garden National Program	‘The Stephanie Alexander Kitchen Garden National Program (SAKNP)’, supports weekly lessons, linking lessons to official curriculum, involvement of community volunteers	Hired garden and kitchen specialists	Primary schools	No; “Engage with social networks and build social capital to enable community ownership”
Wingate, UK, 2017 [41]	Adults; with type 2 Diabetes	Improve diabetes management	Peer support service	Randomized controlled trial of Peer Support In type 2 Diabetes	‘Peer support facilitators’ (PSF) deliver support via 1:1, group or both group and 1:1 peer support (combined)	Community members with Type 2 diabetes trained as PSF	Community centres	No; “Group-based peer support”
Chung, USA, 2018 [38]	African American and Latino adults; with depressive symptoms (PHQ-9 ≥ 10)	Reduce depression and depressive symptoms	Screening and Education programme	Community Engagement and Planning	Multi-sector coalitions to support training and monitoring of local community depression	Council of community members and academics	Faith centres, senior centres, barber shops	No; “Receive support outside of health care in alternative community services”
Visram, UK, 2020 [42]	Adults; Localised to County Durham; Targeting ‘high need populations’: veterans, social isolated older people, mild to moderate mental health issues, manual workers, LGBTQ	Improve prevention health services by integrating existing community services	Signposting service	Wellbeing for Life	Integrated health and wellbeing service by combing one-to-one behaviour change interventions, group wellbeing improvement sessions, volunteer support and capacity building and other community development-related activities	Lay health worker (no formal qualification)	Community hubs and community outreach	Yes
Yeary, USA, 2020 [39]	“Black adults”; Members of at least 1 of 32 selected rural community churches	Prevent diabetes through weight loss	Education programme	WORD	Culturally sensitive adaptation of ‘Diabetes Prevention Programme’; offers small group education sessions	CHW recruited from congregation (selected by local pastor) and trained to deliver service	Churches	No; “[churches] accessibility to underserved groups brings potentials for wide-spread implementation”

The ABAs included: peer support services [*n* = 2: n_IRHSC_ = 1 [34], n_EE_ = 1 [41]]; signposting services [n_EE_ = 1 [42]]; educational programmes without a screening component [*n* = 6: n_IRHSC_ = 1 [32], n_EE_ = 5 [31, 33, 35, 39, 40]]; and educational programmes with a screening component [*n=*2: n_IRC_ = 1 [36], n_EE_ = 1 [37]]. ABAs were delivered by: upskilled staff from within an existing asset [*n* = 4: n_IRC_ = 1 [36], n_IRHSC_ = 1 [32], n_EE_ = 2 [35, 37]]; training public members associated with the asset [n_EE_ = 5 [31, 39–42]]; introducing skilled workers to the asset [*n* = 2: n_IRHSC_ = 1 [34], n_EE_ = 1 [33]]; and by connecting members from across the community network [n_EE_ = 1 [38]].

Each ABA was run across multiple community sites. Approaches included non-religious community venues [*n* = 9: i.e. senior centres [*n* = 4: n_IRC_ = 1 [36], n_EE_ = 3 [32, 35, 37]], bars [n_EE_ = 1 [31]], community centres [*n* = 4: n_IRHSC_ = 2 [32, 34], n_EE_ = 2 [41, 42]], and a primary school [n_EE_ = 1 [33]] or cultural or religious spaces [n_EE_ = 2 [39, 40]]. One study (engaging African American and Latino adults with depressive symptoms) operated across both religious (faith centres) and non-religious spaces (senior centres and barber shops) venues [n_EE_ = 1 [38]]. See Table 2 for further intervention delivery detail.

Targeted populations included: ‘gay and bisexual men’ [n_EE_ = 1 [31]]; individuals ≥ 55 [*n* = 3: n_IRC_ = 1 [36], n_IRHSC_ = 1 [32], n_EE_ = 1 [37]]; school students [n_EE_ = 1 [33]]; ‘Black and minority ethnic groups’ [*n* = 5: n_IRC_ = 1 [36], n_EE_ = 4 [37–40]]; and, adults i) with type 2 Diabetes [n_EE_ = 1 [41]], ii) with depressive symptoms (African Americans and Latinos) [n_EE_ = 1 [38]], iii) up to two years post stroke [n_IRHSC_ = 1 [34]] iv) who are obese [n_EE_ = 1 [35]] and v) residing in North England (County Durham) [n_EE_ = 1 [42]]. County Durham, a mixed rural and urban area, was of interest as resident health is typically poorer than the national average; the service aimed to predominately work with the county’s 30% most deprived communities (no further detail provided on how these communities were defined or identified) [42].

### What designs, methods and outcomes measures have been used to produce cost and economic evidence?

Table 5 summaries the designs, methods and outcome measures used to produce the economic evidence on ABAs.

**Table 5:** Summary of the designs, methods, techniques and outcomes used to produce economic evidence

Study	Comparator	Study type	Primary outcome (Clinical or economic)	Secondary outcomes (Clinical or economic)	Reported costs	Currency (year)	Cost collection approaches
**Reporting only implementation and running costs (IRC)**							
Gitlin, USA, 2012 [36]	Control group: 4 month wait-list	[Single-blind parallel] Randomised trial	PHQ-9	Change in categorical diagnosis	Intervention staff costs (inc travel) Program supervision cost Training costs Programme material costs Adverse events	US$ (2010)	Intervention supervisor and facilitator log
**Reporting implementation and running costs AND health and/or social care related costs (IRHSC)**							
Mayer, USA, 2010 [32]	Post intervention outcomes: between participants and non-participants	Cohort (retrospective)	Total healthcare cost	Inpatient costs; primary care costs; percentage of hospitalisations; number of hospital days	Only total aggregate cost detail	US$ (2005)	From Group Health Cooperative (GHC) (consumer-governed, mixed-model health maintenance organisation with complete cost data for all participants)
Ellis-Hill, UK, 2019 [34]	Control group: standard/typical care	Randomised trial (feasibility study)	Warwick-Edinburgh Mental Well-being Scale (WEMWBS); Hospital Anxiety and Depression Scale (HADS); ICEpop CAPability measure for adults (ICECAP-A)	Rosenberg Self-Esteem Scale (RSES); Medical Outcomes Short Form Health Survey (SF-36 V.1); Head Injury Semantic Differential Scale (HISDS-III)	Intervention staff costs (fixed fee; including travel); Goods (not categorised by consumable and durable); Venue hire cost	£ (2015)	Resource-use form completed by intervention facilitator
**Including an economic evaluation (EE)**							
Kahn, USA, 2001 [31]	Absence of intervention, based on modelled risk reduction	Cohort (prospective)	HIV infection averted QALYs gained		Staff costs (inc travel); Training costs; Goods (durable and consumable); Venue hire; Communication/ networking costs (with community partners)	US$ (2000)	Intervention costs: project ledgers and staff interview Averted health care costs: combing published cost model with recent data on treatment patterns
Stevens, UK, 2002 [40]	Standard/typical care	Panel study	Cost per 1-year quitter Cost per life year saved		Salary cost; Other labour costs; Non-pay costs; Total direct cost; Overheads	£ (unreported)	Actual cost expenditure figures
Krukowski, USA, 2013 [35]	Professionally delivered service	[Cluster] Randomised trial	Cost per kg lost		Assume existing space, staff; Staff costs (time to train lay health worker, production of training and intervention implementation manual); Goods (digital scale and stadiometer, participant recruitment material. calorie and fat counter book for participants, a pedometer, calculator, and lesson binder)	USA$ (unreported)	Unreported
Pizzi, USA, 2014 [37]	Control group: 4 month wait-list	[Single-blind parallel] Randomised trial	QALY as measured by EQ-5D	ICER using HUI-3 derived QALY	“Screening, intervention delivery and supervision”; Health care service use (inpatient; outpatient; medication costs); “Formal care giving and social service use”; Work productivity losses	US$ (2010)	Intervention supervisor and facilitator log
Eckermann, Australia, 2014 [33]	Pre/ post comparison	Cohort	Health outcomes reported in separate publications; Triangulated behaviour change		Staff cost (inc volunteer time); Programme coordinator costs	Au$ (unreported)	Survey completed by Intervention facilitator; Volunteer time at same rate set as kitchen/Garden specialist rate
Wingate, UK, 2017 [41]	Control group: standard/typical care	[Cluster] Randomised trial	Primary/ secondary unspecified: Reference separate publication for complete health outcomes; Key outcomes mentioned: Systolic blood pressure; HbAlc		Hospital staff cost (Nurse, GP Diabetes specialist nurse, Healthcare assistant, Dietician, Consultant); A&E visits; Overnight hospital-stay; Participant out-of-pocket costs (medication, glucose monitoring, cost of medical visits, travel, & other costs)	£ (2013)	Out-of-pocket costs: resource-use form completed by intervention participants; NHS unit costs derived from ‘Unit Costs of Health and Social Care data’
Chung, USA, 2018 (38)	Non-ABA intervention: **‘**Resources for services’: offers technical assistance through webinar and individual primary care practice site visits	[Cluster] Randomised trial	Intervention, planning, training, and service use costs		Screening engagement activities; Training; Goods (consumables); Participant co-production time; Staff; Communication/ networking (with participants); Behavioural health/ depression specific costs (counselling; education; therapy; psychotropic medication; referral) Service-use costs [inpatient hospital night for behavioural health; all emergency room visits; emergency room visits; outpatient primary care visits; social community service visits (family preservation, prisoner re-entry, senior centres, hair salons, exercise clubs); psychotropic medications]	US$ (2010)	Study logs and activity sign-in sheers (wages: time multiplied by hourly wage); Participant-report collected via phone survey; Service use costs: “Consumer Price Index (Hospital and Related Services)” assigned to participant-reported use; Community use costs: estimated from 2010 US Department of Labor national averages of staff wages; Psychotropic medication: match patient reported data to ‘World Health Organisational Daily Defined Dose index’ and compared to 2010 Redbook price data
Visram, UK, 2020 [42]	Forecasted intervention outcomes through behaviour change models	Before and after study	Achieve behaviour change (following intervention: as set in personal health plan)		Staff (time to asset-map, sign-post services); Fixed per participant cost	£ (2014–15)	Trainer data collection and reporting system; Score card completed by intervention facilitator; Data input to ‘Ready Reckoner’ economic model, using 2014/5 cost
Yeary, USA, 2020 [39]	Control group who did not receive additional 12 maintenance sessions	[Cluster] Randomised trial	Cost per kg loss		Staff (volunteer time uncosted) Training Engagement Goods (consumables and durables)	US$ (2014–15)	Budget reports completed throughout intervention

The study designs used to evaluate the ABAs included: cohort studies [*n* = 3: n_IRHSC_ = 1 [32], n_EE_ = 2 [31, 33])]; individual-level randomised controlled trials (RCTs) [*n* = 3: n_IRC_ = 1 [36], n_IRHSC_ = 1 [34] (feasibility trail for larger scale study), n_EE_ = 1 [37]]; cluster RCTs (with ‘clusters’ typically corresponding to the community asset) [n_EE_ = 4 [35, 38, 39, 41]]; a before and after study [n_EE_ = 1 [42]]; and, a panel study [n_EE_ = 1 [40]].

All studies compared, at the minimum, health/wellbeing outcomes against/between:

- post intervention outcomes between participation/ non-participation [n_IRHSC_ = 1 [32]];
- pre- and post- intervention outcomes [n_EE_ = 1 [33]];
- four-month wait-list control group (the control group received the intervention four-months after the intervention arm) [*n* = 2: n_IRC_ = 1 [36], n_EE_ = 1 [37]];
- non-ABA intervention (technical assistance offered through webinar and primary care site visits, ‘traditional’ style intervention delivered for the evaluation) [n_EE_ = 1 [38]];
- healthcare professional delivered service versus ABA lay worker delivered service [n_EE_ = 1: [35]]
- standard/typical care, and [n_EE_ = 4 [34, 39–41]];
- forecasted intervention outcomes through behaviour change models [n_EE_ = 2 [31, 42]].

Primary outcome and secondary outcomes (either health/ wellbeing and/or economic outcomes) were explicitly specified in four papers: in one instance both were cost outcomes [n_IRHSC_ = 1 [32]]; in two instances both were health outcomes [*n* = 2: n_IRC_ = 1 [36], n_IRHSC_ = 1 [34]]; and in a single instance both were measures of cost-effectiveness [‘Incremental cost effectiveness ratio’ (ICER): a summary measure, dividing the difference in total cost by the difference in the chosen measure of health outcome or effect; n_EE_ = 1 [37]]. In all other cases health outcome was reported alongside aggregated cost or within cost-effectiveness measures (not identified as primary or secondary outcomes).

Health outcome measures utilised included: i) PHQ-9 and change in categorical diagnosis [n_IRC_ = 1, [36]]; ii) infections averted and QALY gained [n_EE_ = 1, [31]]; iii) ICECAP-A,^1^ WEMWBS,^2^ HADS^3^; RSES,^4^ SF-36 V.1^5^ and HSDS-111^6^ [n_IRHSC_ = 1, [34]]; iv) achieved behaviour change [n_EE_ = 1 [42]]; v) weight loss [n_EE_ = 1 [39]]; and, vi) EQ-5D and HUI-3- derived QALY [n_EE_ = 1, [37]].

Seven studies report aggregate cost [*n* = 7: n_IRC_ = 1 [36], n_IRHSC_ = 2 [32, 34], n_EE_ = 4 [31, 33, 38, 41]], with five reporting cost within cost-effectiveness measures: ICER [n_EE_ = 2 [37, 40]], ‘value for money’ assessment [n_EE_ = 1 [42]] and ‘cost per kg loss’ [n_EE_ = 2 [35, 39]].

### What is the accrued evidence for the cost and economic impact of ABAs?

Table 6 summaries the accrued evidence for the cost impact of the ABAs. Gitlin 2012 [36] [clustered randomised trial], assigned to IRC, reported cost without comparison. Thus, a conclusion based on this paper, as to whether the ABA is cost-effective cannot be reached. The later published economic evaluation by Pizzi et al. [37] explores the cost-effectiveness of the ABA costed in the Gitlin 2012 [36] (details given below).

**Table 6:** Summary of the accrued evidence for the cost impact of ABAs

Study	Cost-effective?	Cost-effectiveness detail	Resonated with population?	Staff buy-in reported?
**Reporting only implementation and running costs (IRC)**				
Gitlin, USA, 2012 [36]	Not applicable	Resource-intensive home delivery; Resource cost compare favourable to brand name antidepressants	Yes	Yes
**Reporting implementation and running costs AND health and/or social care related costs (IRHSC)**				
Mayer, USA, 2010 [32]	ABA dominant against comparator	Reported non-significant health care cost saving and difference in hospitalisation; Sample participants had significantly lower comorbidity (major driver in hospitalisation) than non-participants	Unreported	Unreported
Ellis-Hill, UK, 2019 [34]	Not applicable	All possible primary outcome measures demonstrate change in favour of ABA intervention; Measures of emotional well-being would be a more relevant study outcome	Yes	Unreported
**Including an economic evaluation (EE)**				
Kahn, USA, 2001 [31]	Cost effective against comparator	Cost per HIV infection averted is far less than the lifetime medical costs of HIV disease; $18,000 or less per infection averted (*excluding* savings from HIV medical costs averted)	Unreported	Unreported
Stevens, UK, 2002 [40]	Cost effectiveness implied (no explicit statement)	ICER = £105 per life year gained (95% CI: £33–391); Modal value: £90 per life year gained; Mean cost per additional 1-year quitter £825 (95% CI: £300–3500)	Unreported	Unreported
Krukowski, USA, 2013 [35]	ABA dominant against comparator	Implementation cost per kilogram lost was $45; Comparable weight loss; LHE delivered service cost almost half as much professionally delivered service; May reached more high-risk individuals	Unreported	Unreported
Pizzi, USA, 2014 [37]	Cost effective against threshold value [Threshold: US $50,000-$100,000/QALY]	Cost-effective treatment for managing depressive symptoms in older African Americans that compares favourably with the cost-effectiveness of previously tested approaches Cost per QALY range of $30,500-$76,500	Yes	Yes
Eckermann, Australia, 2014 [33]	Cost effectiveness implied	Successful health promotion program with high community network impact and return on investment in practice; Multiplier impact on total community activity up to two years was 5.07 ($226,737 against $44,758 invested)	Unreported	Unreported
Wingate, UK, 2017 [41]	Cost effective against comparator	1:1 and group peer support over 8–12 months are cost saving in this setting, largely derived by difference in self-reported utilisations. Long term benefits should be investigated; Overall cost savings of £113.13 per participant per annum	Unreported	Unreported
Chung, USA, 2018 [38]	Comparator dominant against ABA	Conservatively, higher start-up costs in ABA (to engage staff) though reflected increase attendance. Comparator intervention has a time-limited lifetime, longer time horizon may alter result. No significant differences in 12-month service-use costs	Unreported	Yes
Visram, UK, 2020 [42]	Cost effective against threshold value [Threshold: GBP £20,000-£30,000/QALY]	£3,900/QALY gained (comparing favourably with typical UK threshold); Societal value of at least £3.45 for every £1 spent on the service; Model not designed for holistic, multi-component services and therefore possible results represent over- or under- estimation	Unreported	Unreported
Yeary, USA, 2020 [39]	Inconclusive. ABA likely dominant against comparator	$138 per kg lost; In previous studies cost analyses were not conducted separately by race and ethnicity. “Given black typically lose less weight than whites in behavioural weight loss intervention, cost per pound lost may have been considerably high among blacks”	Unreported	Unreported

Category IRHSC consists of two studies. Similarly to Gitlin 2012 [36], Ellis-Hill 2019 [34] [feasibility study] reported cost without a comparison, thus cost-effectiveness cannot be determined [34]. The study notes the lack of suitable comparators within existing literature, precluding easily overcoming the lack of study comparator. Further, the authors make recommendations for more appropriate use of wellbeing measures to support evaluation of intervention impact. Within a retrospective cohort design, Mayer et al. [32] compared participating older adults within senior centres, against non-participating community members. Compared to the non-participants, during the year following the ABA, participants had lower, but non-significant (*P* = 0.58), total health care costs and no difference in hospitalisation. The authors note that ABA participants had significantly higher levels of comorbidity (*P* < 0.001) than the non-participant control. Thus, the comparison may be confounded by the typical increase in hospitalisation seen when comorbidity severity is higher: the ABA impact may be undervalued due to disparity in the study arms. Further, to assess bias, they assessed inclination to use preventative health services – the intervention arm had significantly higher scores, suggesting a stronger tendency to access/use services. Given this, Mayer 2010 [32] is suggestive of a dominant intervention [“A dominant treatment option is one that is both less costly and results in better health outcome than the comparator treatment” [43]].

Nine studies were categorised as EEs, with varying degrees of detail. Using a population-level model Kahn 2001 [31] estimated 5.0 to 6.2 HIV infections averted over five years, at a societal cost of $18,000 or less per infection averted (*excluding* savings from HIV medical costs averted). These authors emphasise different time frames, epidemic scenarios, cost perspectives and modelling inputs lead to a range in cost per infection averted ($4,500 to $46,400) [31]. They believe this compares favourably with other programmes (e.g. Biloxi: $12,000 to $65,000 per HIV case averted) [31]. When savings from HIV medical costs averted are included, the program “eliminates more in HIV medical costs than it costs to implement” (P. 487, [31]).

Stevens [40] used Monte Carlo simulation, estimating intervention costs of £56,986, and reduction in in smoking of 3–7%, resulting in a mean cost of just over £105 per life year gained [40]. Intervention success was noted, in particular, among those not in full time employment. No explicit statement about cost effectiveness was made by the authors. Krukowski [35] compared intervention implementation costs (training; recruitment; materials; ongoing implementation support) of $165 per participant to mean weight loss of 3.7 kg per participant, considered cost-effective compared to a professionally delivered service [35].

Pizzi [37] reported mean incremental costs of $146 per participant per month, with an incremental utility of 0.046 (EQ-5D derived). Base case ICERs, compared to 4-month control, were $64,896 per QALY (EQ-5D) and $36,875 per QALY (HUI-3). Sensitivity analysis yielded cost/QALY range of $20,500-$76,500. The study concludes cost effectiveness compared to threshold values [such thresholds are “the maximum amount a decision-maker is willing to pay for a unit of health outcome” [44]]. Pizzi et al. consider thresholds identified in the literature (US $50,000-$100,000/QALY), and previously reported range of ICERs for pharmacological and neurological depression interventions [37]. Eckermann [33] used an investment multiplier to assess cost effectiveness of an initial government grant, estimating the multiplier impact on total community activity (up to two years) was 5.07 ($226,737 against $44,758 invested). They label this as a successful return on investment (without comparison to any particular threshold), alongside success in health promotion and community network is concluded [33].

Wingate [41] conducted a cost comparison, arguing against a cost effectiveness analysis given no statistically significant difference in condition-specific outcome measure or quality of life assessment between alternatives. Per participant per annum, implementation costs were £13.84, out-of-pocket costs £11.41, but the NHS incurred a cost-saving £138.38 – overall, a *saving of £113.13* [41]. Consequently, the authors conclude the intervention is cost effective, highlighting reductions in self-reported healthcare utilisation. Chung et al. [38] use a cost consequence framework, reporting disaggregated direct and indirect costs across various service sectors. The study comparator had lower overall costs, due to higher start-up costs (to engage and train staff and organisations within the ABA) and no significant differences in 12-month service use cost. However, the authors note capacity was successfully built within community staff, with information shared within the organisation, with potential longer-term benefits not captured in the 12-month evaluation. This omission was common across all studies – none valued the benefit of building capacity/upskilling staff.

Visram [42] conducted a value for money assessment. They estimated total public sector cost savings of £2,406,920 (health gain and cost saving to NHS: £1,477,911; costs offset to NHS from asset mapping and signposting: £798,800; social care: £126,326; criminal justice: £3,883) [42] against service delivery costs of £3,528,894, giving an overall cost of £1,121,974. With an unweighted total health gain of 287.7 QALYs, this results in a cost per QALY of £3,900. Thus, the intervention was deemed cost effective against National Institute for Health and Care Excellence recommended threshold value £20,000-£30,000 per QALY gained [42].

Yeary [39] report per participant mean intervention costs of $348.95 and mean weight loss of 2.53 kg, resulting in a cost of $138 per kg lost. They argue their results indicate cost effectiveness but refrain from giving a concluding statement, due to concerns around the appropriateness of the comparator [there was no ‘true’ control group as both arms received the same core weight loss programme, with the intervention arm receiving a further 12 maintenance sessions]. Mean weight loss was higher in the ABA intervention than a real world setting comparison, based on literature values (an intervention causing an additional 2.1 kg weight loss compared to control) [39]. The authors report challenges to overcoming the lack of intervention comparator through utilising literature values, emphasising that in previous literature costs and outcomes were not reported separately by race: “Given blacks typically loose less weight than white in behavioural weight loss interventions, cost per pound lost may be considerably higher among blacks” (P.2, [39]).

Overall, three papers, representing two ABAs explicitly, report that interventions were well received/ enjoyed/ appreciated by participants, noting them as highly valued or increasing confidence both during intervention participation and beyond (*n* = 3: n_IRC_ = 1 [36], n_IRHSC_ = 1 [34], n_EE_ = 1 [37]). No paper reported this using formal research methods – either qualitatively or through metrics – rather relying on informal research observations. The same studies explicitly mention good engagement and ‘buy-in’ from ABA facilitators.

## Discussion

### What designs, methods and outcomes measures have been used to produce cost and economic evidence?

ABAs in principle seem desirable given pressure to reduce demand on public services and find ways to mitigate inequalities. However, the economic literature about them is extremely limited. A high proportion of included studies suggest that ABAs are cost-effective, a result potentially subject to publication bias, whereby only evidence from ABAs with positive economic findings are published, skewing the published evidence base. If present, as time progresses, publication bias becomes increasingly concerning as “in the presence of publication bias, belief in the relationship increases iteratively with each positive publication” (P.150, [45]). However, with this important caution in mind, we observed: all included ABAs claimed to successfully target and engage underserved, minority or vulnerable populations; such claims would be better substantiated with more formalised capture of participant socioeconomic status. Among the included papers, methods varied, not only in design and comparators, but also in terms of included costs, and outcome measures, likely reflecting the broad scope of ABAs. The prescriptive nature of economic evaluation frameworks may constrain ABA evaluation. Short comings were noted, in both undervaluing of health and wellbeing outcomes, the impact on those beyond the main intervention recipients and short term (time horizon) follow-up of the evaluation.

While studies claimed engagement of underserved populations, none formally reported measured socioeconomic status of participants. Whilst target populations may be assumed, or considered, marginalised, underserved, or residents of more deprived localities, inequality could be better assessed through measurement of socioeconomic status (e.g. income level) or deprivation [e.g. in the UK postcodes (a series alphanumeric characters denoting geographical area used within postal addresses) can be used as a proxy measure for socioeconomic status using established datasets e.g. Index of Multiple Deprivation]. For example, it cannot be known if the more affluent community members (or people who travelling from other more affluent communities) were attending these groups, potentially subverting the ABA aim of engaging the most deprived or marginalised. The importance of this concern will vary by community: if every community member is considered ‘deprived’, engaging any member may be of value. Given the equity improvement focus, more information about the communities in which the ABAs are delivered may help further inform implementation. Reporting of these factors is needed to meaningfully value and prioritise ABAs as a whole and assess their position in inequality improvement – particularly in the context of scare public sector resources. In isolation, ABAs may not fully solve problems of engagement of the underserved but may go some way to resolving them.

Careful consideration should be given to the contextual appropriateness of ABAs. ABAs are useful as they utilise existing resources and can effectively engage people not normally reached. However, it presumes that localities *have* resources upon which to build. Thus, inequalities may be perpetuated were ABAs to be utilised exclusively: communities short of assets may continue to receive no additional interventions. Thus, exclusively relying on ABAs may perpetuate some inequalities. Perhaps, optimally, one might consider ABAs as one ‘tool’ within a wider ‘toolkit’ of approaches for engaging underserved individuals.

Health outcomes were measured within all publications. Ellis-Hill 2019 [34] explored outcome measures for a larger trial and was the only study to include holistic capability measures (e.g. WEMWBS or ICECAP-A). The authors concluded that measures of emotional well-being would be the most relevant study outcome. This adds evidence to the argument that health specific measures (e.g. QALYs) may not fully capture benefits in this setting – ABAs may be better evaluated using more generic measures of wellbeing. Three studies also explicitly reported improvements in participant ‘*enjoyment’*, yet this was not measured, in any capacity, within the utilised costing frameworks. Consequences of social gain (e.g. increasing social confidence to support future engagement or gaining knowledge of services through naturalistic conversations) and emotional well-being improvements should also be considered – holistic capability measures may capture some of these effects.

In ABA evaluation, costs and time horizon need to be carefully considered. Where set-up and training costs are included in intervention delivery costings, ABAs may be disadvantaged if they are compared over short time horizons (1–2 years) – it may be better to consider them one-off set-up costs. However, costing should consider costs of training new staff, maintaining staff, and other ongoing costs – sensitivity analyses may be useful for exploring such points. Further, short time horizons made evidencing long-term impact, and subsequent costs, challenging.

A general challenge of evaluations of preventative or complex interventions, also evidenced here, is capturing the wider life course benefit accrued from engagement with behaviour change interventions. Without a complete retrospective dataset, with a large sample size and an appropriate comparator, it is challenging to definitively assess the impact of such interventions. It is also unknown what would have happened in the absence of the intervention. In some instances (e.g. [31] and [42]), authors made attempts to overcome this by utilising established behaviour change models in their evaluations, including sensitivity analysis to understand the impact of adopted assumptions. However, these models may undervalue ABA contributions if benefits are valued on their contribution/ outcome in the general population (c.f. the earlier consideration of differing weight loss value in white versus black populations).

A key ABA feature, building community capacity, was not captured in costing evaluation frameworks. Given employment is recognised as a ‘social determinant of health’ and a key influencer on overall personal wellbeing, upskilling staff and the wider community, it may well have consequences beyond the intervention focus [46, 47]. Building capacity among staff may offer improved employment opportunities—increasing income—positively contributing to staff’s social determinates of health, potentially reducing their use of health and wellbeing services. However, unmentioned in any included study, is how this may contribute to staff-turnover, perhaps increasing recruitment and training costs.

Relatedly, ABAs are also undervalued where ‘spill over’ effects are not considered, which is of concern since ABAs often impact beyond intervention recipients (e.g. information is shared beyond the sample population to others in the target population or capacity is built among asset staff). Economic evaluation frameworks typically capture the intervention impact on the group of participating individuals. Social return on investment [a “…performance measure similar to ‘return on investment’ but takes a broader societal perspective to valuing cost and benefits. Social and environmental factors are considered, in addition to economic variables to estimate benefit and cost” [48]] may be better suited to captured the wider social and environmental benefits of the intervention, including benefits of building capacity among staff.

Another overlooked cost includes failed engagement of assets – approaching a community asset for an ABA, possibly even developing a unique approach to match their space, population needs, staffing etc., and the proposed intervention not commencing. Publication bias likely plays a role here – if the intervention does *not* commence, then there is little to evaluate and report/publish. Such costs should be considered to ensure realistic and appropriate costing of ABA implementation.

### What is the accrued evidence for the cost and economic impact of ABAs?

Among the identified studies assessing cost-effectiveness, in all but one [38] ABAs were found to be cost-effective, either delivering benefits at a lower cost than an alternative or accruing additional benefits at an acceptable additional cost. However, authors acknowledge the robustness of these conclusions was diminished by the appropriateness of utilised comparators. Inherent to ABA interventions are engaging specific populations, linked to an existing asset in a specific localised area. Consequently, randomisation options are significantly impacted: individual level randomisation will often be infeasible. Therefore, outcome comparisons may be confounded by factors such as population differences. This may be alleviated in part by using analysis that adjusts for such differences: for example, when comparing outcomes between those engaging and not engaging in the intervention, it may be wise to adjust for inclination to engage with services, as discussed in Mayer 2010 [32]. However, this can only address *known and measured* confounders. Some studies utilised a comparison between participant and non-participants (e.g. [32]): however, the validity of this design may be decreased with some ABA interventions, where ‘participation’ may not always be clear-cut (for example in information sharing interventions) – statistical contamination may occur between comparison groups.

Some evaluations had no comparator. Among these, some sought to compare outcomes to values from literature to demonstrate beneficial impacts. However, two studies noted they were inhibited from doing so because of a lack of relevant literature. This reinforces the more general consideration of the need for improved representation of underserved populations in literature. This general goal may be supported through disaggregating study data by population group (e.g. sub-group analysis).

Some ABAs promote access to other health and wellbeing improvement services, employment, and job services. From the current evidence base it is unclear whether ABA participation does indeed support access to secondary services. A key motivation for utilising ABAs is to help people from disengaged populations to take preventative action, potentially reducing their future use of other health and social care resources. By failing to measure the impact on wider health and wellbeing services, the value of ABAs may be either i) grossly undervalued or ii) missing costly impacts on wider services. Such considerations are also impacted by the adopted time horizon: health and social service costs may rise in the short term (e.g. increased use of screening services).

As the emphasis on community intervention grows, ABAs, and their economic evidence, should be contextualised to the current economic and political climate. For example, the publication gap 2020–23 may be a consequence of the COVID-19 pandemic. Community assets suffered because of government-imposed restrictions preventing in-person gatherings, diminishing community participation. As we move toward to a post lockdown era, entering a cost-of-living crisis, there is significantly higher demand on community assets (such as libraries or foodbanks). Consequently, adopting an ABA and ‘asking’ more of community assets should be considered with caution. For example, in a number of the ABAs evaluated, there was no mention of venue or room hire costs; with current rising costs, the ability of assets to ‘absorb’ increased costs (e.g. hiring a room for longer to deliver a ABA) is likely significantly reduced. Consequently, policy makers should consider if it is sustainable, or indeed ethical, to ‘push’ the responsibility of ABAs on to already stretched assets.

In conducting this review, we found many of the excluded screened papers made mention of ABAs being ‘economically efficient’ without formally evaluating this domain. It is important such claims are validated with evidence, so that the reality of implementation is known. Further, publications generally lacked details on implementation strategies, resulting in the possible exclusion of some papers which may have been an ABA. As the weight of evidence of community intervention increases, implementation strategies should be routinely reported, and consistent terminology employed. This allows for an inference of community impact, in the instances where economic impact fails to fully capture the contextualised *social value*.

## Conclusions

Economic literature on ABAs is extremely limited. ABAs seem to be a promising way to successfully target and engage underserved, minority or vulnerable populations, and may not result in a higher net cost when compared to other approaches to health and wellbeing improvement. The included ABAs represent a broad range of approaches, with methods of evaluation varying widely, not only in design and comparators, but also in terms of included costs, and outcome measures. The current use of economic evaluation methodologies do not capture well the full impacts of ABAs, likely both undervaluing health and wellbeing outcomes (i.e. capacity building, holistic health improvements, increased secondary health and wellbeing service use, and long-term impact on health) and staff capacity building, as well as underestimating delivery costs (i.e. venue higher and failed asset engagement). Current economic evidence struggles due to lack of appropriate intervention comparator, made challenging by the hyper-local nature of ABAs.

In health economics generally, interventions are typically considered as mutually exclusive occurrences, however in the context of community health, and specifically ABAs, this approach typically misses the economic reality of embedding services. The economics of the surrounding services, mechanisms of information sharing, and collaboration underpin the success of assets and ABAs. The economic evidence, and evaluations in general, would benefit from further detail to help ensure more nuanced and sophisticated application of ABAs. Further evidence is needed to determine the cost-effectiveness of ABAs.

### Supplementary Information

**Supplementary Material.**

## Data Availability

Data sharing is not applicable to this article as no datasets were generated or analysed during the current study.

## Abbreviations

ABAs: Asset-based approaches
CASP: Critical Appraisal Skills Programme
CENTRAL: Cochrane Centre Register of Controlled Trials
CHW: Community Health Workers
EE: Economic evaluation
EoE: East of England
EQ-5D: EuroQol five dimension questionnaire
HADS: Hospital Anxiety and Depression Scale
HDS-111: Head Injury Semantic Differential Scale
ICE-CAP-A: ICEpop CAPability measure for adults
NHS: National Health Service
PHQ-9: Patient Health Questionnaire – 9
PPI: Patient and public involvement
PRISMA: Preferred Reporting Items for Systematic Reviews and Meta-Analyses
QALY: Quality adjusted life-years
RSES: Rosenberg Self-Esteem Scale
SF-36 V.1: Medical Outcomes Short Form Health Survey
WALYs: Wellbeing adjusted life years
WELBYs: Wellbeing adjusted life years
WEMWBS: Warwick-Edinburgh Mental Well-being Scale

## Footnotes

1 ICEpop CAPability measure for adults.

2 Warwick-Edinburgh Mental Well-being Scale.

3 Hospital Anxiety and Depression Scale.

4 Rosenberg Self-Esteem Scale.

5 Medical Outcomes Short Form Health Survey.

6 Head Injury Semantic Differential Scale.
