# Supplementary material for: Cost and economic evidence for asset-based approaches to health improvement and their evaluation methods: a systematic review
Source: BMC Public Health. 2024 Mar 15;24:814. doi: 10.1186/s12889-024-18231-4 (PMC10941621; doi:10.1186/s12889-024-18231-4)
Supplement: Supplementary file 1 — Supplementary Material. [file 12889_2024_18231_MOESM1_ESM.docx]

Cost and economic evidence for asset-based approaches to health improvement and their evaluation methods: a systematic review

**Supplementary Material**

**Supplementary Material 1**

("Health*"[tiab] OR "Social"[tiab] OR "Wellbeing"[tiab])

AND

("cost*"[ti] OR "return on investment"[ti] OR "SROI"[ti] OR "programme budget"[ti] OR "budget report"[ti] OR "budget analysis"[ti] OR "marginal analysis"[ti] OR "social capital"[ti] OR "willingness to pay"[ti] OR "WTP"[ti] OR "Economic*"[ti])

AND

("asset*"[tiab] OR "ABA"[tiab] OR "co-production"[tiab] OR "community development"[tiab] OR "community engagement"[tiab] OR "community empowerment"[tiab] OR "self-care"[tiab] OR "capabilities"[tiab] OR "communitarian claims"[tiab] OR "resilience"[tiab] OR "ABCD"[tiab] OR "social ecological model"[tiab] OR "participatory approach*"[tiab] OR "CBPR"[tiab] OR "informal care"[tiab] OR "peer support"[tiab] OR "salutogenesis"[tiab] OR "upstream"[tiab] OR "social prescri*"[tiab] OR "intervention*"[tiab] OR "health promotion"[tiab] OR "wellbeing officer"[tiab] OR "lay worker"[tiab] OR "community health worker"[tiab] OR "CHW"[tiab] OR "upskill"[tiab] OR "capacity"[tiab] OR "training"[tiab])

AND

("asset*"[tiab] OR "local"[tiab] OR "communit*"[tiab] OR "Neighborhood"[tiab] OR "Neighbourhood"[tiab])

**Supplementary Material 2**

Headings for data extraction

- Study
- Population
- Aim
- Intervention type
- Intervention title
- Intervention detail
- Individual delivering service
- Community setting
- Named as ABA?
- Comparator
- Study type
- Primary outcome
- Secondary outcome
- Reported cost
- Cost currency (year)
- Cost collection approach
- Cost effective? Y/N
- Cost effective detail
- Resonate with population?
- Staff buy-in reported?

**Supplementary Material 3**

**CASP Cohort**

**CASP RCT**

**CASP EE**
